# MERIT40-dependent recruitment of tankyrase to damaged DNA and its implication for cell sensitivity to DNA-damaging anticancer drugs

**DOI:** 10.18632/oncotarget.26312

**Published:** 2018-11-09

**Authors:** Keiji Okamoto, Tomokazu Ohishi, Mika Kuroiwa, Shun-ichiro Iemura, Tohru Natsume, Hiroyuki Seimiya

**Affiliations:** ^1^ Division of Molecular Biotherapy, Cancer Chemotherapy Center, Japanese Foundation for Cancer Research, Koto-ku, Tokyo, Japan; ^2^ Laboratory of Molecular Target Therapy of Cancer, Department of Computational Biology and Medical Sciences, Graduate School of Frontier Sciences, The University of Tokyo, Koto-ku, Tokyo, Japan; ^3^ Molecular Profiling Research Center for Drug Discovery, National Institute of Advanced Industrial Science and Technology, Koto-ku, Tokyo, Japan; ^4^ Current address: Institute of Microbial Chemistry (BIKAKEN), Numazu, Shizuoka, Japan; ^5^ Current address: Translational Research Center, Fukushima Medical University, Fukushima, Japan

**Keywords:** tankyrase, MERIT40, DNA damage response, DNA repair, cancer chemotherapy

## Abstract

Tankyrase, a member of the poly(ADP-ribose) polymerase (PARP) family, regulates various intracellular responses, such as telomere maintenance, Wnt/β-catenin signaling and cell cycle progression through its interactions with multiple target proteins. Tankyrase contains a long stretch of 24 ankyrin repeats that are further divided into five subdomains, called ANK repeat clusters (ARCs). Each ARC works as an independent ligand-binding unit, which implicates tankyrase as a platform for multiple protein-protein interactions. Furthermore, tankyrase distributes to various intracellular loci, suggesting potential distinct but yet unidentified physiological functions. To explore the novel functions of tankyrase, we performed liquid chromatography-mass spectrometry analysis and identified the BRE-BRCC36-MERIT40 complex, a regulator of homologous recombination, as tankyrase-binding proteins. Among the complex components, MERIT40 was directly associated with tankyrase via a tankyrase-binding consensus motif, as previously reported. In X-ray-irradiated non-small cell lung cancer cells, tankyrase localized to DNA double-stranded break sites in a MERIT40-dependent manner. MERIT40 knockdown increased the cell sensitivity to X-ray, whereas the wild-type, but not the tankyrase-unbound mutant, MERIT40 rescued the phenotype of the knockdown cells. Tankyrase inhibitors, such as G007-LK and XAV939, increased the cellular sensitivity to X-ray irradiation and anticancer drugs that induce DNA double-stranded breaks. These observations suggest that tankyrase plays a role in the DNA damage repair response and implicates a potential therapeutic utility of tankyrase inhibitors in combination treatments with DNA-damaging anticancer drugs.

## INTRODUCTION

Tankyrase is a member of the poly(ADP-ribose) polymerase (PARP) family of proteins that regulates various intracellular processes by adding poly(ADP-ribose) chains, or poly(ADP-ribosyl)ating (PARsylating), to target proteins using NAD^+^ as a substrate [1, 2]. For example, tankyrase interacts with and PARsylates telomeric repeat-binding protein 1 (TRF1), a negative regulator of telomerase-mediated telomere elongation. PARsylated TRF1 is then dissociated from telomeres, which allows access of telomerase and subsequent telomere elongation. Thus, tankyrase functions as a positive regulator of telomere length in telomerase-positive human cells [1]. Tankyrase is also involved in regulating Wnt/β-catenin signaling. In the Wnt signaling pathway, Axin forms the destruction complex with APC, glycogen synthase kinase 3β (GSK-3β) and casein kinase to phosphorylate and degrade β-catenin, causing reduction of β-catenin-dependent gene expression [3–5]. Tankyrase binds and PARsylates cytoplasmic Axin, which promotes the subsequent degradation of PARsylated Axin in an E3 ubiquitin ligase RNF146-dependent manner [6, 7]. Tankyrase thus functions as a positive regulator of the Wnt signaling pathway, and the development of tankyrase inhibitors as potential anticancer drugs for Wnt-driven cancer is under investigation [8]. Tankyrase also plays a role in regulating the mitotic spindle, centrosome maturation and cancer cell invasion by its interaction with NuMA, MIKI and TNKS1BP1, respectively [9–11]. Tankyrase contains multiple ANK repeat clusters (ARCs), each of which can bind the above-mentioned proteins via the consensus motif Rxx(G/P/A/C)(D/x)G. A recent proteomic analysis identified a variety of tankyrase-binding proteins with a broad range of cellular distributions and functions [12]. Together these findings demonstrate that tankyrase is a multifunctional protein with several yet unidentified functions.

DNA double-stranded breaks (DSBs) are one of the most severe forms of DNA damage that occurs by both external and internal factors, such as ionizing radiation, genotoxic chemicals and reactive oxygen species, among others. To protect cells from the deleterious effect of DSBs, DSBs are rapidly recognized by DNA damage response (DDR) proteins and immediately repaired. The MRE11-RAD50-NBS1 complex is the first sensor of DSBs and recruits ATM to phosphorylate histone H2AX at serine 139 (γH2AX) [13, 14]. γH2AX is recognized by MDC1, which is also phosphorylated by ATM, followed by the recruitment of RNF8 and RNF168, which conjugates a lysine 63 (K63)-linked ubiquitin chain on H2A/H2AX. These steps are required for recruitment of the downstream proteins involved in the regulation of DNA damage repair pathway, such as 53BP1 and the BRCA1 complex [15–20]. There are two main DNA repair pathway for DSBs: non-homologous end joining (NHEJ) and homologous recombination (HR). While NHEJ directly connects two broken ends after a minor DNA resection, HR uses the complementary strand at the sister chromatid to synthesize the correct sequence during the S/G2 phase of the cell cycle. In HR, the BRCA1-CtIP complex is recruited to DSBs for DNA resection in a 5′ to 3′ direction to expose single-stranded overhangs, which promote strand invasion and subsequent DNA synthesis [21]. In addition to the BRCA1-CtIP complex, BRCA1 also interacts with Abraxas1/CCDC98, BRE/BRCC45, MERTI40/NBA1, BRCC36 and RAP80, to form the BRCA1-A complex. The BRCA1-A complex is recruited to DSBs at a late phase of DDR through the ubiquitin-interacting motif domain of RAP80 that is directly associated with the K63-linked ubiquitin chain [22, 23]. BRE and MERIT40 stabilize this complex and maintain the localization of the BRCA1-A complex at DSBs, where the BRCA1-A complex removes K63 ubiquitination by the de-ubiquitinase BRCC36 and suppresses excessive DNA resection and subsequent HR [24–26].

In this study, we performed liquid chromatography-mass spectrometry (LC-MS) analysis and identified the MERIT40-BRE-BRCC36 complex as tankyrase-binding proteins. We found that tankyrase is localized to DSBs through its interaction with MERIT40, and pharmacological inhibition of tankyrase sensitized human lung cancer cells to DNA-damaging anticancer agents. These observations suggest a role for tankyrase in DDR and a potential use for tankyrase inhibitors in combination with anticancer drugs.

## RESULTS

### Tankyrase binds to MERIT40 via the tankyrase-binding motif

**Figure 1:**
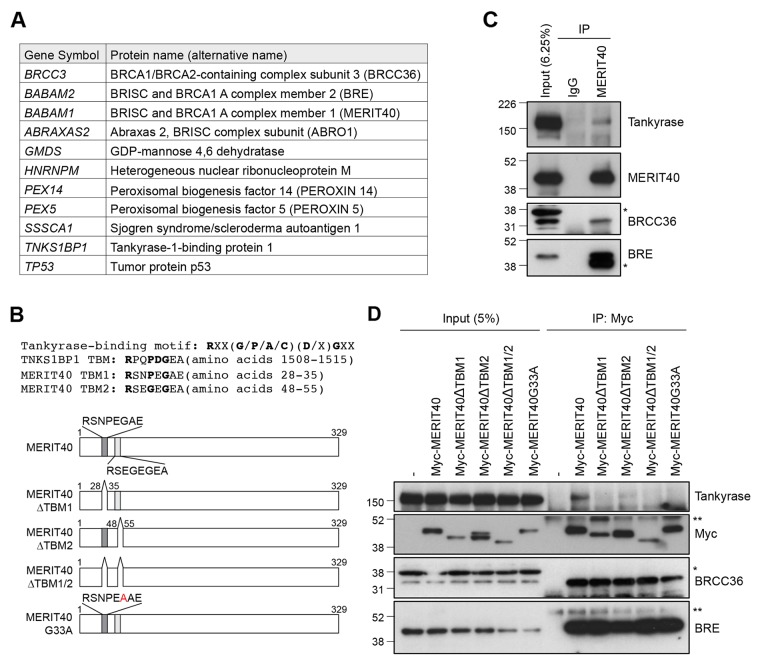
Tankyrase is associated with the BRE-BRCC36-MERIT40 complex **(A)** List of tankyrase-binding proteins identified by liquid chromatography/mass spectrometry (LC-MS) analysis. FLAG-tagged tankyrase was overexpressed in HEK293T cells, and the cell lysates were immunoprecipitated with anti-FLAG antibody. The immunocomplexes were subjected to LC-MS analysis. **(B)** *Top*: tankyrase-binding motif (TBM) in TNKS1BP1 and MERIT40. *Bottom*: schematic diagram of MERIT40 mutants used in this study. Dark and light gray boxes indicate putative tankyrase-binding motifs. **(C)** Association of endogenous tankyrase and MERIT40 in A549 cells. A549 cells were immunoprecipitated (IP) with anti-MERIT40 antibody and subjected to western blot analysis with the indicated antibodies. Normal mouse immunoglobulin G (IgG) was used as negative control. **(D)** Association of tankyrase with Myc-tagged MERIT40 constructs. Myc-tagged MERIT40 was transiently expressed in HeLa cells and immunoprecipitated with anti-Myc antibody, followed by western blotting with anti-tankyrase, Myc, BRCC36 and BRE antibodies. Single and double asterisks indicate non-specific band and IgG heavy chain, respectively.

To identify novel tankyrase-binding proteins, we immunoprecipitated transiently expressed FLAG-tagged tankyrase in HEK293T cells and performed LC-MS analysis of the immunocomplexes. The results identified 11 tankyrase-interacting proteins, including TNKS1BP1, a well-established tankyrase-binding protein (Figure 1A) [27]. The potential 11 tankyrase-binding partners included BRCC3/BRCC36, BRE/BABAM2 and MERIT40/BABAM1, which are components of the BRCA1-A complex. Furthermore, ABRO1/ABRAXAS2, which forms another complex with BRCC36-BRE-MERIT40 in the cytoplasm called the BRISC1 complex [28–30], was also in the identified protein list. MERIT40 is a 329 amino acid protein that possesses two tankyrase-binding motifs (TBM): RSNPEGAE at amino acids 28–35 (TBM1) and RSEGEGEA at amino acids 48–55 (TBM2) (Figure 1B) [31, 32]. We examined the interaction between MERIT40 and tankyrase in A549 cells and confirmed that endogenous tankyrase was co-immunoprecipitated with MERIT40 (Figure 1C). BRCC36 and BRE were also detected by the immunoprecipitation with anti-MERIT40 antibody (Figure 1C), suggesting that tankyrase was another member of the BRE-BRCC36-MERIT40 complex.

To determine which motif is involved in binding to tankyrase, we established HeLa cells that stably express Myc-tagged MERIT40 mutants lacking one or both TBMs (MERIT40ΔTBM1, MERIT40ΔTBM2 or MERIT40ΔTBM1/2) (Figure 1B and 1D). Immunoprecipitation using anti-Myc antibody showed that while endogenous tankyrase bound wild-type Myc-tagged MERIT40, binding was reduced to MERIT40ΔTBM2 and completely lost with MERIT40ΔTBM1 and MERIT40ΔTBM1/2. Furthermore, a MERIT40 point mutant, in which glycine residue in TBM1 (G33), an essential amino acid to associate with tankyrase, was substituted by alanine (MERIT40-G33A), also lost the ability to bind tankyrase (Figure 1B and 1D). In contrast, all MERIT40 mutants maintained binding with BRCC36 and BRE, suggesting that the MERIT40 mutants were structurally functional [33–35]. These data demonstrate that MERIT40 binds to tankyrase mainly via the TBM1.

### Tankyrase inhibitors sensitize lung cancer cells to X-ray irradiation

**Figure 2:**
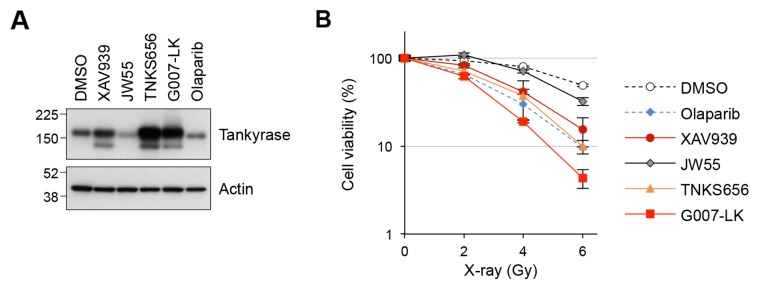
Tankyrase inhibitors sensitize A549 cells to X-ray irradiation **(A)** Tankyrase accumulation upon treatment with tankyrase inhibitors. A549 cells were treated with tankyrase inhibitors (XAV939, JW55, TNKS656 and G007-LK) or a PARP1/2 inhibitor (olaparib) at 3 μM for 16 h and then examined by western blot analysis. Actin served as a loading control. **(B)** Effects of tankyrase inhibitors on A549 cell sensitivity to X-ray irradiation. Cells were treated with the indicated tankyrase inhibitors or the PARP1/2 inhibitor olaparib at 3 μM for 16 h, followed by X-ray irradiation at 2, 4 or 6 Gy. After a 10-day incubation, the numbers of colonies were quantified. Data were normalized with the colony numbers at 0 Gy, in which cell viability was defined as 100%. Three independent experiments were performed and each experiment was performed in triplicate.

Our LC-MS results identified BRCC36, BRE and MERIT40 as tankyrase-binding proteins; these proteins are components of the BRCA1-A complex, which regulates HR in response to DSBs. To examine if tankyrase plays a role in the DDR pathway, we investigated the response to X-ray irradiation in A549 cells treated with small-molecule tankyrase inhibitors, XAV939, JW55, TNKS656 and G007-LK [6, 36–38]. Tankyrase inhibitors cause accumulation of tankyrase protein due to prevention of auto-PARsylation, which otherwise leads to ubiquitin-dependent degradation of tankyrase. Indeed, tankyrase protein levels were elevated in A549 cells treated with 3 μM of XAV939, TNKS656 or G007-LK, confirming the pharmacodynamic effects of these inhibitors (Figure 2A). By contrast, 3 μM olaparib, a PARP1/2 inhibitor, did not increase the protein level of tankyrase. Treatment with another tankyrase inhibitor, JW55, at 3 μM resulted in degradation of tankyrase, as previously reported [38].

Next, A549 cells pre-treated with tankyrase or PARP1/2 inhibitors at 3 μM for 16 h were irradiated with 2, 4 or 6 Gy of X-ray, and colony formation assays were performed after 10 days of incubation. The results showed that XAV939, TNKS656 and G007-LK sensitized the cells to X-ray exposure compared to DMSO (Figure 2B). As a positive control, olaparib, a known sensitizer of ionizing radiation [39], enhanced the cell sensitivity to X-ray exposure. The effect of JW55 was only minimal, if any, which corresponded to the lack of accumulation of tankyrase protein in these cells. Except for JW55, the sensitizing effects of the tankyrase inhibitors were comparable to or even higher than that of olaparib. These observations suggest that tankyrase plays a role in the DDR pathway.

### Potential requirement of MERIT40-mediated tankyrase localization to DNA DSBs for DNA repair

**Figure 3:**
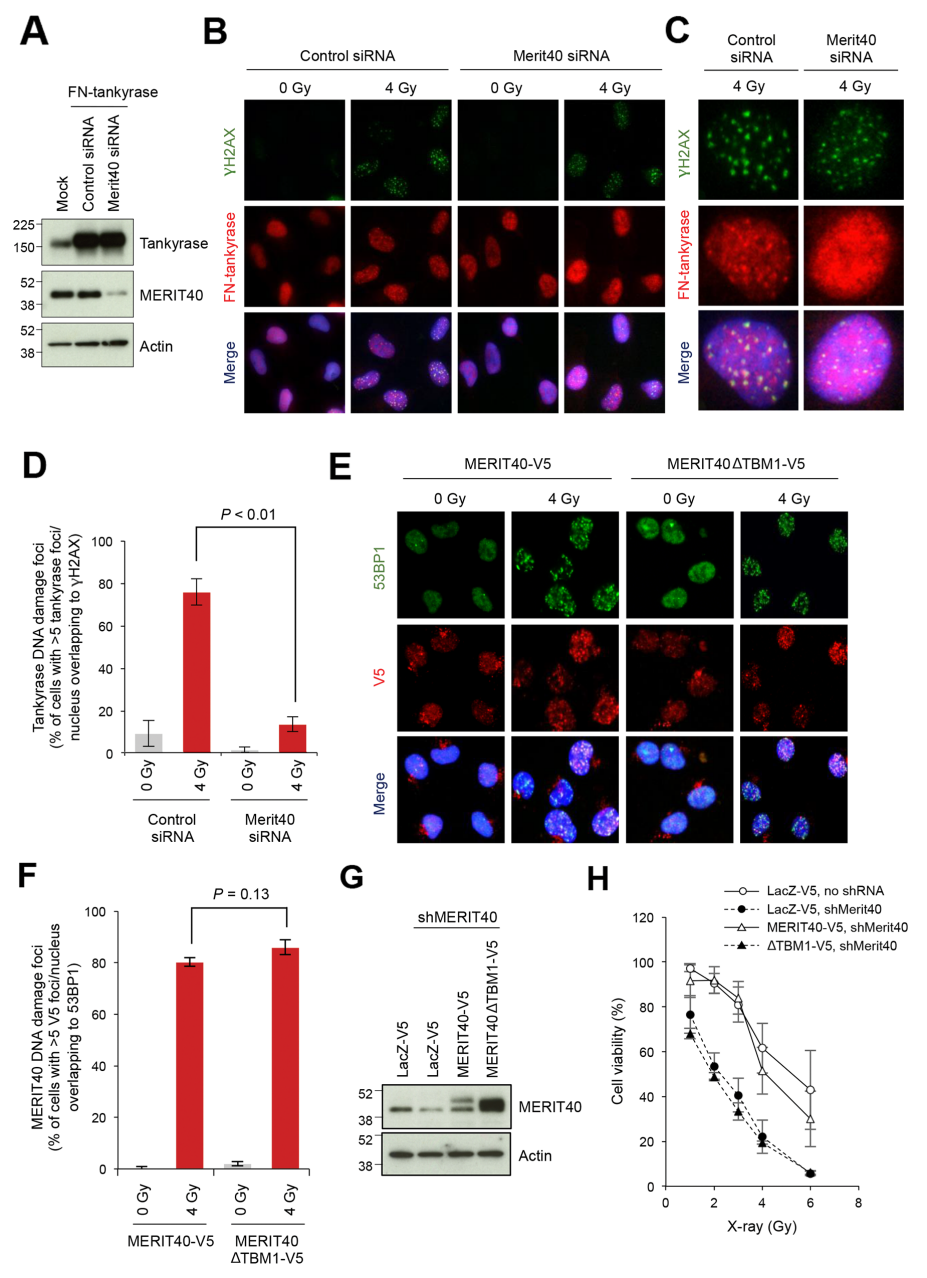
Disruption of MERIT40-mediated recruitment of tankyrase to DSBs decreases the viability of X-ray-irradiated A549 cells **(A)** Western blot of A549 cells with ectopically expressed and nuclear localized tankyrase and depletion of MERIT40. Cells were transfected with an expression vector for FN-tankyrase and MERIT40 siRNA as indicated. After 48 h, western blot analysis was performed with the indicated antibodies. β-actin served as loading control. **(B)** Indirect immunofluorescence staining. Cells treated as in A were irradiated with 4 Gy of X-ray, incubated for 6 h, and subjected to immunofluorescence staining with anti-γH2AX (green) and anti-tankyrase (red) antibodies. Nuclei were counterstained with DAPI (blue). **(C)** Magnified views of representative cells in B. **(D)** The percentages of the cells with more than five tankyrase foci/nucleus that overlapped with γH2AX foci shown in B and C were quantified. Statistical analysis was performed by student's two-tailed *t-*test. **(E)** Foci formation of MEIT40ΔTBM1 mutant. V5-tagged, siRNA-resistant MERIT40 (wild-type and ΔTBM1 mutant) was stably expressed in A549 cells. These cells were transfected with MERIT40 siRNA to deplete only endogenous MERIT40. After 48 h, cells were irradiated with 4 Gy of X-ray, followed by incubation for 6 h. Cells were subjected to immunofluorescence staining with anti-53BP1 (green) and anti-V5 (red) antibodies. Nuclei were counterstained with DAPI (blue). **(F)** The percentages of the cells with more than five V5 foci/nucleus that overlapped with 53BP1 foci in E were quantified. Statistical analysis was performed by Student's two-tailed *t-*test. **(G)** Western blot analysis of A549 cells that stably expressed MERIT40 shRNA and either MERIT40-V5 or MERIT40ΔTBM1-V5. Cells, in which shRNA-resistant V5-tagged MERIT40 (wild-type or ΔTBM1 mutant) or LacZ was stably expressed, were infected with lentivirus for MERIT40 shRNA. After selection, cell lysates were prepared and subjected to western blot analysis with the indicated antibodies. β-actin served as loading control. **(H)** X-ray sensitivity of the cells in (G) Cells were irradiated by indicated doses of X-ray. After a 10-day incubation, the numbers of colonies were quantified. Data were normalized with the colony numbers at 0 Gy, in which cell viability was defined as 100%. Three independent experiments were performed and each experiment was performed in triplicate.

We next examined whether tankyrase localizes to DSBs. In our preliminary experiments, it was difficult to monitor endogenous nuclear tankyrase by immunofluorescence staining because of the relatively low abundance of the nuclear tankyrase and intense signals of cytoplasmic tankyrase (data not shown). Therefore, to monitor the localization of tankyrase in the nuclei, we established A549 cells that stably expressed FLAG-tagged and nuclear localizing signal-fused tankyrase (FN-tankyrase) and confirmed its protein expression and nuclear localization (Figure 3A, 3B). This tankyrase construct has been previously used for studying the nuclear functions of tankyrase [40–42]. Co-immunostaining for FN-tankyrase and γH2AX, a DNA damage marker, in X-ray-irradiated cells revealed that tankyrase formed nuclear foci that were co-localized to γH2AX foci (Figure 3B–3D). Importantly, when MERIT40 was depleted by siRNA in FN-tankyrase stable cells (Figure 3A), the percentage of cells with the FN-tankyrase foci were reduced from 76.0% to 13.7% in the irradiated cells (Figure 3B–3D).

To examine whether the tankyrase-MERIT40 interaction is required for MERIT40 localization to DSBs, we examined irradiation-induced foci formation in cells expressing the MERIT40 mutant lacking tankyrase binding. We established A549 cells stably expressing MERIT40 or MERIT40ΔTBM1 tagged with V5. To exclude the influence of endogenous MERIT40, these cells were then transfected with MERIT40 siRNA targeting the 5′-untranslated region, which exogenous MERIT40 and MERIT40ΔTBM1 did not possess. Cells were then irradiated with X-ray at 4 Gy (Figure 3E). The wild-type MERIT40 formed nuclear foci that co-localized with 53BP1, a marker of DSBs, indicating that the V5 epitope tag did not affect the localization and damage response of MERIT40. Furthermore, MERIT40ΔTBM1 was still able to form foci on DSBs, similar to the wild-type MERIT40 (Figure 3E and 3F). To reinforce the idea that the absence of tankyrase-MERIT40 interaction exacerbates the deleterious effect of DNA damage, we examined X-ray sensitivity of A549 cells, in which the endogenous MERIT40 was stably knocked down by shRNA, and the shRNA-resistant V5-tagged MERIT40 exogene (wild-type or ΔTBM1 mutant) was introduced (Figure 3G). As shown in Figure 3H, MERIT40 knockdown increased the cell sensitivity to X-ray, whereas the wild-type but not ΔTBM1 mutant MERIT40 rescued the phenotype of the knockdown cells. Together these observations suggest that while the tankryase-MERIT40 interaction is dispensable for MERIT40 localization to DSBs, MERIT40-dependent recruitment of tankyrase to DSBs is required to execute proper DNA repair.

### Combination effect of a tankyrase inhibitor and DNA-damaging agents

**Figure 4:**
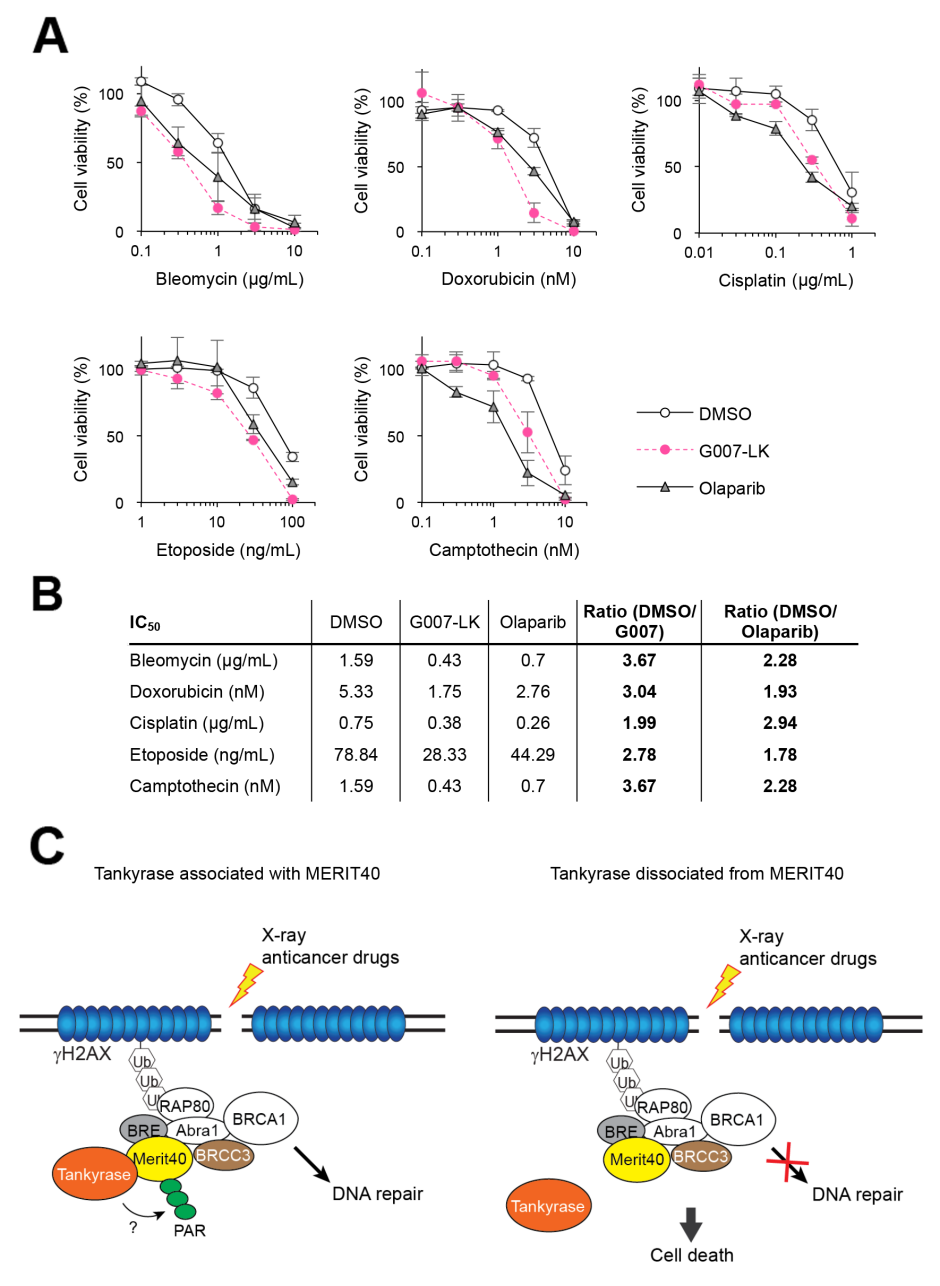
Tankyrase inhibitor enhances the growth inhibitory effects of DNA-damaging anticancer drugs **(A)** A549 cells were treated with DNA-damaging anticancer drugs (bleomycin, doxorubicin, cisplatin, etoposide and camptothecin) at various concentrations in the presence or absence of 3 μM G007-LK or olaparib. After a 10-day cultivation, colonies were quantitated and normalized with the colony numbers from cells treated with either DMSO, G007-LK or olaparib alone, in which cell viability was defined as 100%. Three independent experiments were performed and each experiment was performed in triplicate. **(B)** The IC_50_ values of each drug treatment in A is shown in the table. The ratios of IC_50_ values in the combination treatment of DNA-damaging anticancer drugs with G007-LK or olaparib compared with the anticancer drugs with DMSO are shown in the right column. **(C)** A schematic model of potentiated sensitivity to DNA damage by tankyrase inhibition. *Left*: when DNA double-strand break (DSB) is created by X-ray or anticancer drugs, tankyrase forms a complex with MERIT40-BRE-BRCC36 through direct association with MERIT40. Then, tankyrase-mediated PARsylation plays a role to facilitate the DNA repair process. *Right*: inhibition of either tankyrase-MERIT40 association or tankyrase PARP activity causes dysfunction in DNA repair process, resulting in deleterious effects, such as cell death.

From the above observations, we speculated that tankyrase inhibitors might have a combination effect with anticancer drugs that induce DNA damage. A549 cells pretreated with G007-LK or olaparib as a positive control were treated with anticancer drugs, such as bleomycin, doxorubicin, cisplatin, etoposide and camptothecin, which induce DNA DSBs. Cisplatin and camptothecin induce inter-strand crosslink and single-stranded breaks, respectively, and both cause DSBs. As shown in Figure 4A, while A549 cells showed a dose-dependent inhibition in growth by treatment with each of the cytotoxic agents, the sensitivity was potentiated by the addition of G007-LK or olaparib. While the potentiating effects of G007-LK and olaparib were almost comparable for each anticancer drug, the ratios of IC_50_ values indicate that G007-LK enhanced the cytotoxicity of bleomycin, doxorubicin, etoposide and camptothecin more potently than olaparib did (Figure 4B). Meanwhile, G007-LK enhanced the effect of cisplatin less efficiently than olaparib. Similar results were obtained by another tankyrase inhibitor XAV939 (Supplementary Figure 1). These observations support the notion that tankyrase is functionally involved in the DDR or DNA repair pathway.

## DISCUSSION

In this study, we demonstrated a potential role of tankyrase in the DDR machinery. Our LC-MS analysis and immunoprecipitation data showed that tankyrase was associated with the BRE-BRCC36-MERIT40 complex (or the BRCA1-A complex) via MERIT40. In the BRCA1-A complex, MERIT40 directly binds to BRE and Abraxas1, which functions as a platform for the other components, BRCC36, RAP80 and BRCA1 [33–35, 43]. Our LC-MS did not detect Abraxas1, RAP80 or BRCA1 as tankyrase-binding proteins because we used FLAG-tagged tankyrase, which does not accumulate in the nuclei of cells [44]. This may be the reason that all the components of the cytoplasmic BRISC complex (BRE, BRCC36, MERIT40, ABRO1) were identified as tankyrase-interacting proteins, which result was similar to the previous report [45], instead of those of the nuclear BRCA1-A complex.

MERIT40 was previously identified as a tankyrase-binding protein through *in silico* analysis [31]. Our immunoprecipitation experiments confirmed the endogenous tankyrase-MERIT40 interaction mainly via the first consensus tankyrase-binding site in MERIT40 at amino acids 28–35 (TBM1). Furthermore, we found that tankyrase was recruited to DSBs through its association with MERIT40 and tankyrase-MERIT40 interaction was necessary for the enhanced viability of X-ray-irradiated A549 cells. These data suggest that tankyrase has a potential role in the regulation of the DDR machinery, possibly for HR, as a component of the BRCA1-A complex (Figure 4C). Indeed, our results showed that tankyrase inhibitors potentiated the sensitivity of A549 cells to X-ray and DNA-damaging agents.

A previous study showed that MERIT40 is PARsylated by tankyrase, although the PARsylation level was weaker than those of other tankyrase-binding proteins, such as Disc1, STRIATIN, Fat4 and BCR [31]. Tankyrase-mediated PARsylation of some proteins, such as TRF1, Axin and PTEN, target these proteins for degradation by the ubiquitin-proteasome pathway [7, 46–48]. For example, FN-tankyrase overexpression downregulates TRF1 protein level in a proteasome-dependent manner [42, 49]. By contrast, we did not observe downregulation of MERIT40 in tankyrase-overexpressing cells (Figure 3A). This would be reminiscent of TNKS1BP1, another tankyrase-binding protein, which is PARsylated by tankyrase but not downregulated in tankyrase-overexpressing cells [10, 27]. One possibility is that PAR chains in the tankyrase-MERIT40 complexes work as a scaffold to promote DNA repair as PARP-1/2-derived PAR chains play such a role. Alternatively, considering that the BRCA1-A complex suppresses excessive DNA resection and HR by being recruited at the late phase of DDR, tankyrase-mediated PARsylation may destabilize MERIT40 to maintain HR at an adequate level. Further, inhibition of tankyrase may overstabilize the BRCA1-A complex and cause prevention of HR. As mentioned above, however, overexpression of FN-tankyrase did not reduce the endogenous level of MERIT40 in A549 cells (Figure 3A).

A previous study showed that siRNA-mediated knockdown of tankyrase downregulates the catalytic subunit of DNA-dependent protein kinase (DNA-PKcs) and promotes telomere recombination [50]. This indicates that tankyrase PARP activity is required for the stability of DNA-PKcs protein, which is functionally involved in NHEJ, and repression of telomere recombination. A recent report showed that E3 ligase RNF8 and de-ubiquitinase BRCC36-containing BRISC complex control the stability of tankyrase in a cell cycle-dependent manner [45]. These observations suggest that tankyrase and the proteins involved in DDR pathway or DNA repair machineries regulate each other. Of note, tankyrase also binds MDC1, a mediator of both HR and NHEJ, and this interaction is required for tankyrase recruitment to DSBs and efficient HR [51]. Intriguingly, however, tankyrase inhibition by XAV939 does not affect the efficiency of HR [51]. Further examination will be required to unravel the precise mechanism for tankyrase-mediated regulation of HR.

Since tankyrase positively regulates Wnt/β-catenin signaling, tankyrase inhibitors have been expected to be novel anticancer therapeutics, especially for Wnt-driven colorectal cancer [2]. However, this treatment strategy still faces obstacles, as prolonged exposure to tankyrase inhibitors may lead to intestinal toxicity due to inhibition of Wnt/β-catenin signaling and proliferation of intestinal crypt cells [36]. In the present study, we found that tankyrase inhibitors sensitized cancer cells to DNA-damaging anticancer drugs. This finding could be applied to a combination therapy of tankyrase inhibitors and DNA-damaging anticancer drugs. In this case, it may be possible to decrease doses of both tankyrase inhibitors and DNA-damaging anticancer drugs to help minimize the side effects of the drugs.

## MATERIALS AND METHODS

### Cell culture and RNAi

Human lung adenocarcinoma A549 were obtained from Dr. Takao Yamori in 1998. Human cervical adenocarcinoma HeLa I.2.11 cells were obtained from Dr. Susan Smith in 2001; cells were re-authenticated by short tandem repeat (STR) analysis (BEX, Tokyo, Japan) in 2018. Both cell lines were cultured in Dulbecco's modified Eagle's Medium with low glucose and 10% heat-inactivated fetal bovine serum at a humidified atmosphere with 5% CO_2_.

MERIT40 siRNA was designed by Thermo Fisher Scientific (Waltham, MA). The target sequence of MERIT40 siRNA and shRNA is 5′-GTTTGTCATGGATAATTTTTT-3′ and 5’-CACCTTCTTGTGCAAGGAAGT-3’, respectively. The control siRNA was also purchased from Thermo Fisher Scientific (#4390843).

### Vector constructs and virus infection

The cDNAs for wild-type MERIT40, MERIT40ΔTBM1, MERIT40ΔTBM2 and MERIT40ΔTBM1/2 were amplified by PCR and subcloned into the pBabe-Myc-puro vector. MERIT40G33A vector was generated from pBabe-Myc-MERIT40 using QuikChange II XL Site-Directed Mutagenesis Kit (#200522, Agilent Technologies, Santa Clara, CA). To produce retroviruses, GP2-293 cells were transfected with these vectors and pVSV-G using polyethylenimine (PEI) MAX (#24765-1, Polysciences, Inc., Warrington, PA). A549 or HeLa cells were infected with the retroviruses and selected with 1 μg/mL puromycin for 3 days. To produce lentivirus, the MERIT40 cDNAs were inserted into the pLenti6/V5-Dest vector (V49610, Thermo Fisher Scientific) according to manufacturer's instructions. Lentiviruses were produced by co-transfection of HEK293FT cells with ViraPower Lentiviral Packaging Mix (K497500, Thermo Fisher Scientific) using PEI MAX. After 48 h, A549 or HeLa cells were infected with the lentiviruses and selected with 10 μg/mL blasticidin for 3 days.

### Liquid chromatography-mass spectrometry (LC-MS)

HEK293T cells were transfected with the FLAG-tagged tankyrase plasmid using Lipofectamine 2000 (Lifetechnologies, Carlsbad, CA) according to the manufacturer's protocol. The cells were lysed with lysis buffer (20 mM Hepes, pH 7.5, 150 mM NaCl, 50 mM NaF, 1 mM Na_3_VO_4_, 0.5% digitonin, 1 mM PMSF, 5 μg/ml leupeptin, 5 μg/ml aprotinin, and 3 μg/ml pepstatin A). After centrifugation, the supernatant was incubated with anti-FLAG M2-agarose beads (Sigma-Aldrich, St. Louis, MO) and the beads were washed with wash buffer (10 mM Hepes, pH 7.5, 150 mM NaCl, and 0.1% Triton X-100). The immunoprecipitants were eluted with a FLAG peptide (0.5 mg/ml; Sigma-Aldrich) dissolved in wash buffer and digested with lysyl endopeptidase (Lys-C; Wako Chemicals USA). The immunoprecipitated proteins were analyzed by a direct nanoflow liquid chromatography/tandem mass spectrometry system coupled to a time-of-flight mass spectrometer (Q-STAR XL; AB Sciex, Foster City, CA), as described previously [52].

### Immunoprecipitation and western blot analysis

Cells were harvested and lysed in TNE lysis buffer [60 mM Tris-HCl pH 7.5, 150 mM NaCl, 1 mM EDTA, 0.5% Nonidet P-40 and protease inhibitor cocktail (25955-11, Nacalai Tesque, Kyoto, Japan)]. Lysates were centrifuged, and the supernatants were incubated with anti-Myc (1:200, #2276S, Cell Signaling Technology, Danvers, MA) or MERIT40 antibodies (5 μg/mL, A302-515A, Bethyl Laboratories, Montgomery, TX) and rotated overnight at 4°C. Dynabeads Protein G (#10003D, Thermo Fisher Scientific) were then added and samples were further rotated for 1 h at 4°C. Beads were washed with TNE lysis buffer, and the co-precipitated proteins were eluted by boiling in SDS sample buffer (50 mM Tris-HCl pH 6.8, 2% SDS, 10% glycerol and 4% 2-mercaptoethanol). Proteins were subjected to SDS-PAGE and transferred on polyvinylidene fluoride membranes. Western blot analysis was performed with anti-Myc (1:1000, #2276S, Cell Signaling Technology), tankyrase (0.4 μg/mL, sc-8337, Santa Cruz Biotechnology, Dallas, TX), MERIT40 (1 μg/mL, Bethyl Laboratories), BRCC36 (1 μg/mL, #4331, ProSci, Inc., Poway, CA), BRE (0.4 μg/mL, sc-376453, Santa Cruz Biotechnology) and β-actin (1:20000, A5441, Sigma-Aldrich) primary antibodies. Anti-rabbit IgG (conformation Specific) (HRP-conjugate) (1:2000, #5127, Cell Signaling Technology), anti-mouse IgG for IP (HRP) (1 μg/mL, ab131368, Abcam, Cambridge, UK) and anti-rabbit and mouse IgG HRP linked whole antibodies (NA934V and NA931V, respectively, GE Healthcare, Little Chalfont, United Kingdom) were used as secondary antibodies. Signals were detected using an ECL Western Blotting Detection System (RPN2106, GE Healthcare) or Pierce Western Blotting Substrate Plus (NCI32132, Thermo Fisher Scientific) and developed to film using an X-OMAT 2000 processor (Kodak, Hemel Hempstead, United Kingdom).

### Colony formation assays

A549 cells seeded in 6-well plates (500 cells/well) were treated with tankyrase inhibitors [XAV939 (#3748, Tocris Bioscience, Bristol, United Kingdom), JW55 (#4514, Tocris Bioscience), TNKS656 (provided by Dr. Fumiyuki Shirai, RIKEN) and G007-LK (S7239, Selleck Chemicals, Houston, TX) or a PARP1/2 inhibitor (olaparib, S1060, Selleck Chemicals)] at 3 μM for 16 h, treated with X-ray irradiation at various doses and then cultured for 10 days. In another assay, cells pretreated with or without 3 μM XAV939, 3 μM G007-LK or 3 μM olaparib for 16 h were treated with various concentrations of DNA-damaging anticancer drugs [bleomycin (BML-AP302, Enzo Biochem, Farmingdale, NY), doxorubicin (D1515, Sigma-Aldrich), etoposide (BML-GR307, Enzo Biochem), cisplatin (ALX-400-40, Enzo Biochem) or camptothecin] in the presence or absence of 3 μM XAV939 and cultured for 10 days. Colonies were stained with 0.5% crystal violet/25% methanol and quantified by colony analyzer CA-7II (System Science Co., Tokyo, Japan) or ImageJ software (National Institute of Health, Bethesda, MD).

### Immunofluorescence microscopy

Immunofluorescence staining was performed as previously described [53]. The following primary antibodies were used: anti-53BP1 (1:250, #4937, Cell Signaling Technology), anti-phospho-histone H2A.X Ser139 (0.1 μg/mL, #05-636, Millipore, Burlington, MA), tankyrase (1 μg/mL, sc-8337, Santa Cruz Biotechnology) and V5 (0.24 μg/mL, R960-25, Thermo Fisher Scientific). Alexa Fluor 594 or 488-conjugated anti-mouse or rabbit IgG (4 μg/ml, A-11032, A-11029, A-11037 and A-11034, Life Technologies) were used as secondary antibodies. Images were acquired using an Olympus IX71 microscope with a DP80 digital camera (Olympus, Tokyo, Japan) and analyzed by cellSens software (Olympus).

### Statistical analysis

All quantitative data were shown as mean ± standard deviation. For statistical analysis, student's two-tailed *t*-test was performed to assess the significance between compared samples. *P*-value less than 0.05 was considered statistically significant.

## SUPPLEMENTARY MATERIALS FIGURES



## Abbreviations

DDR: DNA damage response
FN-tankyrase: FLAG-tagged and nuclear localizing signal-fused tankyrase
HR: homologous recombination
LC-MS: liquid chromatography-mass spectrometry
NHEJ: nonhomologous end joining
PARP: poly(ADP-ribose) polymerase
PARsylation: poly(ADP-ribosyl)ation
TBM: tankyrase-binding motif

## References

[1] Smith S, Giriat I, Schmitt A, de Lange T (1998). Tankyrase, a poly(ADP-ribose) polymerase at human telomeres. Science.

[2] Bai P (2015). Biology of poly(ADP-ribose) polymerases: the factotums of cell maintenance. Mol Cell.

[3] Behrens J, Jerchow BA, Wurtele M, Grimm J, Asbrand C, Wirtz R, Kuhl M, Wedlich D, Birchmeier W (1998). Functional interaction of an axin homolog, conductin, with beta-catenin, APC, and GSK3beta. Science.

[4] Hart MJ, de los Santos R, Albert IN, Rubinfeld B, Polakis P (1998). Downregulation of beta-catenin by human Axin and its association with the APC tumor suppressor, beta-catenin and GSK3 beta. Curr Biol.

[5] Rubinfeld B, Albert I, Porfiri E, Fiol C, Munemitsu S, Polakis P (1996). Binding of GSK3beta to the APC-beta-catenin complex and regulation of complex assembly. Science.

[6] Huang SM, Mishina YM, Liu S, Cheung A, Stegmeier F, Michaud GA, Charlat O, Wiellette E, Zhang Y, Wiessner S, Hild M, Shi X, Wilson CJ (2009). Tankyrase inhibition stabilizes axin and antagonizes Wnt signalling. Nature.

[7] Zhang Y, Liu S, Mickanin C, Feng Y, Charlat O, Michaud GA, Schirle M, Shi X, Hild M, Bauer A, Myer VE, Finan PM, Porter JA (2011). RNF146 is a poly(ADP-ribose)-directed E3 ligase that regulates axin degradation and Wnt signalling. Nat Cell Biol.

[8] Riffell JL, Lord CJ, Ashworth A (2012). Tankyrase-targeted therapeutics: expanding opportunities in the PARP family. Nat Rev Drug Discov.

[9] Chang W, Dynek JN, Smith S (2005). NuMA is a major acceptor of poly(ADP-ribosyl)ation by tankyrase 1 in mitosis. Biochem J.

[10] Ohishi T, Yoshida H, Katori M, Migita T, Muramatsu Y, Miyake M, Ishikawa Y, Saiura A, Iemura SI, Natsume T, Seimiya H (2017). Tankyrase-binding protein TNKS1BP1 regulates actin cytoskeleton rearrangement and cancer cell invasion. Cancer Res.

[11] Ozaki Y, Matsui H, Asou H, Nagamachi A, Aki D, Honda H, Yasunaga S, Takihara Y, Yamamoto T, Izumi S, Ohsugi M, Inaba T (2012). Poly-ADP ribosylation of Miki by tankyrase-1 promotes centrosome maturation. Mol Cell.

[12] Li X, Han H, Zhou MT, Yang B, Ta AP, Li N, Chen J, Wang W (2017). Proteomic analysis of the human tankyrase protein interaction network reveals its role in pexophagy. Cell Rep.

[13] Burma S, Chen BP, Murphy M, Kurimasa A, Chen DJ (2001). ATM phosphorylates histone H2AX in response to DNA double-strand breaks. J Biol Chem.

[14] Uziel T, Lerenthal Y, Moyal L, Andegeko Y, Mittelman L, Shiloh Y (2003). Requirement of the MRN complex for ATM activation by DNA damage. EMBO J.

[15] Doil C, Mailand N, Bekker-Jensen S, Menard P, Larsen DH, Pepperkok R, Ellenberg J, Panier S, Durocher D, Bartek J, Lukas J, Lukas C (2009). RNF168 binds and amplifies ubiquitin conjugates on damaged chromosomes to allow accumulation of repair proteins. Cell.

[16] Goldberg M, Stucki M, Falck J, D’Amours D, Rahman D, Pappin D, Bartek J, Jackson SP (2003). MDC1 is required for the intra-S-phase DNA damage checkpoint. Nature.

[17] Huen MS, Grant R, Manke I, Minn K, Yu X, Yaffe MB, Chen J (2007). RNF8 transduces the DNA-damage signal via histone ubiquitylation and checkpoint protein assembly. Cell.

[18] Lou Z, Minter-Dykhouse K, Wu X, Chen J (2003). MDC1 is coupled to activated CHK2 in mammalian DNA damage response pathways. Nature.

[19] Mailand N, Bekker-Jensen S, Faustrup H, Melander F, Bartek J, Lukas C, Lukas J (2007). RNF8 ubiquitylates histones at DNA double-strand breaks and promotes assembly of repair proteins. Cell.

[20] Stewart GS, Panier S, Townsend K, Al-Hakim AK, Kolas NK, Miller ES, Nakada S, Ylanko J, Olivarius S, Mendez M, Oldreive C, Wildenhain J, Tagliaferro A (2009). The RIDDLE syndrome protein mediates a ubiquitin-dependent signaling cascade at sites of DNA damage. Cell.

[21] Chen L, Nievera CJ, Lee AY, Wu X (2008). Cell cycle-dependent complex formation of BRCA1.CtIP.MRN is important for DNA double-strand break repair. J Biol Chem.

[22] Kim H, Chen J, Yu X (2007). Ubiquitin-binding protein RAP80 mediates BRCA1-dependent DNA damage response. Science.

[23] Sobhian B, Shao G, Lilli DR, Culhane AC, Moreau LA, Xia B, Livingston DM, Greenberg RA (2007). RAP80 targets BRCA1 to specific ubiquitin structures at DNA damage sites. Science.

[24] Dong Y, Hakimi MA, Chen X, Kumaraswamy E, Cooch NS, Godwin AK, Shiekhattar R (2003). Regulation of BRCC, a holoenzyme complex containing BRCA1 and BRCA2, by a signalosome-like subunit and its role in DNA repair. Mol Cell.

[25] Hu X, Kim JA, Castillo A, Huang M, Liu J, Wang B (2011). NBA1/MERIT40 and BRE interaction is required for the integrity of two distinct deubiquitinating enzyme BRCC36-containing complexes. J Biol Chem.

[26] Hu Y, Scully R, Sobhian B, Xie A, Shestakova E, Livingston DM (2011). RAP80-directed tuning of BRCA1 homologous recombination function at ionizing radiation-induced nuclear foci. Genes Dev.

[27] Seimiya H, Smith S (2002). The telomeric poly(ADP-ribose) polymerase, tankyrase 1, contains multiple binding sites for telomeric repeat binding factor 1 (TRF1) and a novel acceptor, 182-kDa tankyrase-binding protein (TAB182). J Biol Chem.

[28] Cooper EM, Cutcliffe C, Kristiansen TZ, Pandey A, Pickart CM, Cohen RE (2009). K63-specific deubiquitination by two JAMM/MPN+ complexes: BRISC-associated Brcc36 and proteasomal Poh1. EMBO J.

[29] Cooper EM, Boeke JD, Cohen RE (2010). Specificity of the BRISC deubiquitinating enzyme is not due to selective binding to Lys63-linked polyubiquitin. J Biol Chem.

[30] Patterson-Fortin J, Shao G, Bretscher H, Messick TE, Greenberg RA (2010). Differential regulation of JAMM domain deubiquitinating enzyme activity within the RAP80 complex. J Biol Chem.

[31] Guettler S, LaRose J, Petsalaki E, Gish G, Scotter A, Pawson T, Rottapel R, Sicheri F (2011). Structural basis and sequence rules for substrate recognition by tankyrase explain the basis for cherubism disease. Cell.

[32] Sbodio JI, Chi NW (2002). Identification of a tankyrase-binding motif shared by IRAP, TAB182, and human TRF1 but not mouse TRF1. NuMA contains this RXXPDG motif and is a novel tankyrase partner. J Biol Chem.

[33] Feng L, Huang J, Chen J (2009). MERIT40 facilitates BRCA1 localization and DNA damage repair. Genes Dev.

[34] Shao G, Patterson-Fortin J, Messick TE, Feng D, Shanbhag N, Wang Y, Greenberg RA (2009). MERIT40 controls BRCA1-Rap80 complex integrity and recruitment to DNA double-strand breaks. Genes Dev.

[35] Wang B, Hurov K, Hofmann K, Elledge SJ (2009). NBA1, a new player in the Brca1 A complex, is required for DNA damage resistance and checkpoint control. Genes Dev.

[36] Lau T, Chan E, Callow M, Waaler J, Boggs J, Blake RA, Magnuson S, Sambrone A, Schutten M, Firestein R, Machon O, Korinek V, Choo E (2013). A novel tankyrase small-molecule inhibitor suppresses APC mutation-driven colorectal tumor growth. Cancer Res.

[37] Shultz MD, Cheung AK, Kirby CA, Firestone B, Fan J, Chen CH, Chen Z, Chin DN, Dipietro L, Fazal A, Feng Y, Fortin PD, Gould T (2013). Identification of NVP-TNKS656: the use of structure-efficiency relationships to generate a highly potent, selective, and orally active tankyrase inhibitor. J Med Chem.

[38] Waaler J, Machon O, Tumova L, Dinh H, Korinek V, Wilson SR, Paulsen JE, Pedersen NM, Eide TJ, Machonova O, Gradl D, Voronkov A, von Kries JP (2012). A novel tankyrase inhibitor decreases canonical Wnt signaling in colon carcinoma cells and reduces tumor growth in conditional APC mutant mice. Cancer Res.

[39] Calabrese CR, Almassy R, Barton S, Batey MA, Calvert AH, Canan-Koch S, Durkacz BW, Hostomsky Z, Kumpf RA, Kyle S, Li J, Maegley K, Newell DR (2004). Anticancer chemosensitization and radiosensitization by the novel poly(ADP-ribose) polymerase-1 inhibitor AG14361. J Natl Cancer Inst.

[40] Ohishi T, Hirota T, Tsuruo T, Seimiya H (2010). TRF1 mediates mitotic abnormalities induced by Aurora-A overexpression. Cancer Res.

[41] Seimiya H, Muramatsu Y, Ohishi T, Tsuruo T (2005). Tankyrase 1 as a target for telomere-directed molecular cancer therapeutics. Cancer Cell.

[42] Smith S, de Lange T (2000). Tankyrase promotes telomere elongation in human cells. Curr Biol.

[43] Wang B, Matsuoka S, Ballif BA, Zhang D, Smogorzewska A, Gygi SP, Elledge SJ (2007). Abraxas and RAP80 form a BRCA1 protein complex required for the DNA damage response. Science.

[44] Smith S, de Lange T (1999). Cell cycle dependent localization of the telomeric PARP, tankyrase, to nuclear pore complexes and centrosomes. J Cell Sci.

[45] Tripathi E, Smith S (2017). Cell cycle-regulated ubiquitination of tankyrase 1 by RNF8 and ABRO1/BRCC36 controls the timing of sister telomere resolution. EMBO J.

[46] DaRosa PA, Wang Z, Jiang X, Pruneda JN, Cong F, Klevit RE, Xu W (2015). Allosteric activation of the RNF146 ubiquitin ligase by a poly(ADP-ribosyl)ation signal. Nature.

[47] Li N, Zhang Y, Han X, Liang K, Wang J, Feng L, Wang W, Songyang Z, Lin C, Yang L, Yu Y, Chen J (2015). Poly-ADP ribosylation of PTEN by tankyrases promotes PTEN degradation and tumor growth. Genes Dev.

[48] Bhardwaj A, Yang Y, Ueberheide B, Smith S (2017). Whole proteome analysis of human tankyrase knockout cells reveals targets of tankyrase-mediated degradation. Nat Commun.

[49] Chang W, Dynek JN, Smith S (2003). TRF1 is degraded by ubiquitin-mediated proteolysis after release from telomeres. Genes Dev.

[50] Dregalla RC, Zhou J, Idate RR, Battaglia CL, Liber HL, Bailey SM (2010). Regulatory roles of tankyrase 1 at telomeres and in DNA repair: suppression of T-SCE and stabilization of DNA-PKcs. Aging (Albany NY).

[51] Nagy Z, Kalousi A, Furst A, Koch M, Fischer B, Soutoglou E (2016). Tankyrases promote homologous recombination and check point activation in response to DSBs. PLoS Genet.

[52] Natsume T, Yamauchi Y, Nakayama H, Shinkawa T, Yanagida M, Takahashi N, Isobe T (2002). A direct nanoflow liquid chromatography-tandem mass spectrometry system for interaction proteomics. Anal Chem.

[53] Okamoto K, Bartocci C, Ouzounov I, Diedrich JK, Yates JR, Denchi EL (2013). A two-step mechanism for TRF2-mediated chromosome-end protection. Nature.

