# Supplementary material for: MERIT40-dependent recruitment of tankyrase to damaged DNA and its implication for cell sensitivity to DNA-damaging anticancer drugs
Source: Oncotarget. 2018 Nov 9;9(88):35844–55. doi: 10.18632/oncotarget.26312 (PMC6254674; doi:10.18632/oncotarget.26312)
Supplement: Supplementary file 1 [file oncotarget-09-35844-s001.pdf]

MERIT40-dependent recruitment of tankyrase to damaged DNA and its implication for cell sensitivity to DNA-damaging anticancer drugs

SUPPLEMENTARY MATERIALS

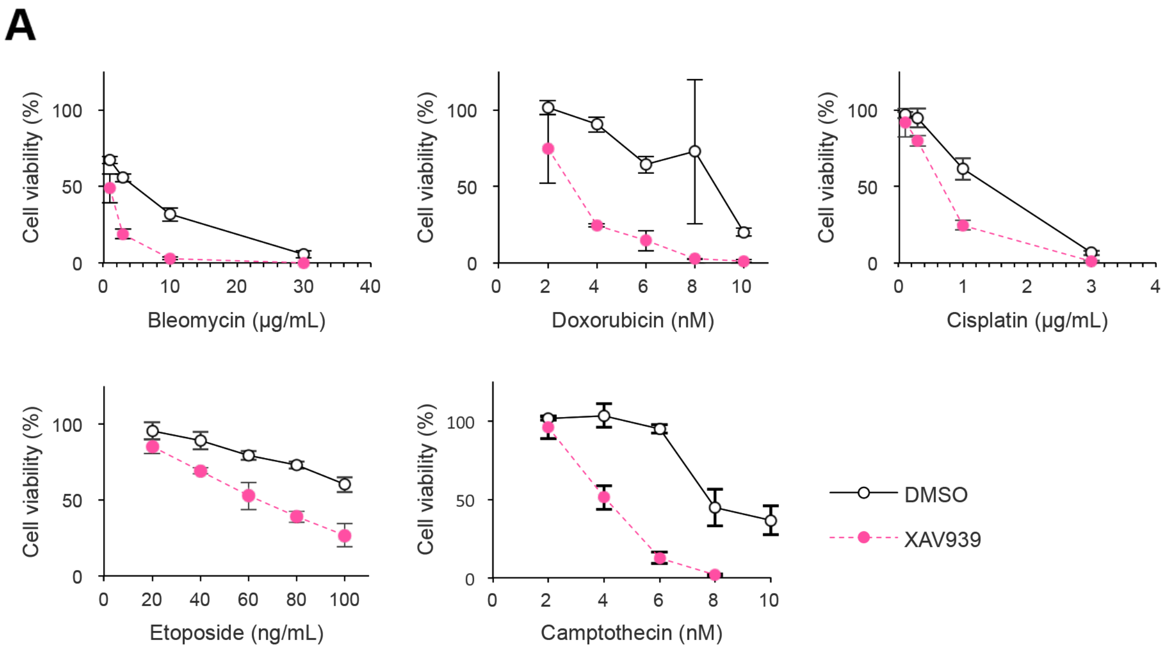

B

IC_{50}	DMSO	XAV939	Ratio (DMSO/ XAV)
Bleomycin (µg/mL)	4.63	0.97	**4.77**
Doxorubicin (nM)	8.86	2.99	**2.96**
Cisplatin (µg/mL)	1.42	0.68	**2.08**
Etoposide (ng/mL)	>100	64.01	**N/A**
Camptothecin (nM)	7.79	4.06	**1.92**

Supplementary Figure 1: Tankyrase inhibitor XAV939 enhances the growth inhibitory effects of DNA-damaging anticancer drugs. (A) A549 cells were treated with DNA-damaging anticancer drugs (bleomycin, doxorubicin, cisplatin, etoposide and camptothecin) at various concentrations in the presence or absence of 3 µM XAV939. After a 10-day cultivation, colonies were quantitated and normalized with the colony numbers from cells treated with either DMSO or XAV939 alone, in which cell viability was defined as 100%. Three independent experiments were performed and each experiment was performed in triplicate. **(B)** The IC_{50} values of each drug treatment in A is shown in the table. The ratios of IC_{50} values in the combination treatment of DNA-damaging anticancer drugs with XAV939 compared with the anticancer drugs with DMSO are shown in the right column.
